# Insights into the genome of Azotobacter sp. strain CWF10, isolated from an agricultural field in Central India

**DOI:** 10.1099/acmi.0.000930.v4

**Published:** 2025-01-28

**Authors:** Arindam Roy, Anwesha Ghosh, Yash, Prateeksha Mehra, Sumit Roy, Punyasloke Bhadury

**Affiliations:** 1 Integrative Taxonomy and Microbial Ecology Research Group, Department of Biological Sciences, Indian Institute of Science Education and Research Kolkata, Mohanpur, Nadia, 741246, West Bengal, India; 2 World Wide Fund for Nature-India, Pirojsha Godrej Building, 172B Lodhi Road, Lodhi Estate, New Delhi, 110003, India; 3 Centre for Climate and Environmental Studies, Indian Institute of Science Education and Research Kolkata, Mohanpur, Nadia, 741246, West Bengal, India

**Keywords:** *Azotobacter*, nitrogen, plant growth-promoting genes, siderophores

## Abstract

*Azotobacter* sp. strain CWF10, an aerobic gram-negative, oval-shaped and motile bacterium, was isolated from the lateritic agricultural soil of Madhya Pradesh, India. The draft genome of the isolate is 5.7 Mb in size, consisting of 14 contigs with 65.09% G+C content. Average nucleotide identity (94.66%) and digital DNA–DNA hybridization (62%) calculation with the closest reference strains underpin the bacterium as a potential novel species. The bacterium has a plethora of plant growth-promoting genes that point towards the potential ability to enhance available nitrogen and biosynthesis of folic acid, among others. Siderophores such as vibrioferrin and crochelin A are also present in the genome and are known to regulate iron uptake. Overall, mining the genome of *Azotobacter* sp. strain CWF10 has revealed the potential of this strain for application in regenerative agriculture and sustaining soil health.

## Data Summary

The whole-genome sequence data are available on NCBI (BioProject Accession Number PRJNA1159111 and BioSample Accession Number SAMN43561090).

## Introduction

Biological nitrogen fixation is a crucial ecological phenomenon and is largely driven by free-living as well as symbiotic microbes [1]. The fixation of atmospheric nitrogen in the soil, as an alternative to Haber–Bosch industrial production of nitrogen fertilisers for boosting agricultural productivity, is more economical and environmentally sustainable. Members of the genus *Azotobacter*, belonging to the family Pseudomonadaceae, are ubiquitous across soil ecosystems globally. Since the 20th century, various strains of *Azotobacter* have been exploited as bioinoculants to solve the problem of nitrogen deficiency-associated plant growth [2].

## Methods

The strain CWF10 was isolated from nutrient-deficient agricultural soil in the Chhindwara district of Madhya Pradesh, India. Briefly, 1 g of soil (top 0–5 cm) was serially diluted 10^6^ times, and 100 µl was plated on nitrogen-free agar medium (glucose 20 g l^–1^, CaCO_3_ 20 g l^–1^, K_2_HPO_4_ 1 g l^–1^, MgSO_4_.7H_2_O 0.5 g l^–1^, agar 15 g l^–1^, pH 7.2) [3,4]. The pale brown colony was subsequently inoculated in liquid medium and incubated under the following conditions (30 °C, 80 r.p.m.). Genomic DNA was extracted following the published methods [5,6]; quality and quantity were ascertained following the published protocol [7]. To undertake phylogeny based on 16S rRNA, FC27 and RC1492 [8] were used. Whole-genome sequencing was undertaken on a MinION platform using Oxford Nanopore Technologies sequencing chemistry following published protocols [7]. Porechop (v0.2.0) was used to remove adapters from long reads. Subsequently, assembly into the draft genome was undertaken using Flye (v2.9.3) [9]. The quality of the assembled genome was verified using QUAST (v5.2.0) [10] and CheckM [11]. The genome map was visualized using Proksee [12]. FastME 2.1.6.1 [13] was used to calculate inter-genomic distance as well as for SPR post-processing [14]. To ascertain the closest taxonomic affiliation, 16S rRNA sequence taxonomic relatives were retrieved from databases (GenBank/ENA/DDBJ) using the blastn tool and aligned using clustalW [15]. The maximum likelihood-based best-fit nucleotide substitution model for aligned sequences was inferred by jModeltest (v2.1.6) [16]. Further, an ML tree was constructed using the Tamura–Nei model (BIC=8175.2576) with 1000 bootstrap replicates in mega11 [17]. Digital DNA–DNA hybridization (dDDH) was calculated using Genome-to-Genome Distance Calculator [18], and the average nucleotide identity (ANI) was calculated using the ANIb algorithm [19] on the JSpeciesWS server [20] for demarcation of pecies rank (e.g. novel). BlastKOALA [21] was used for functional annotation of the assembled genome [22]. Antibiotic resistance genes were annotated and identified using the Resistance Gene Identifier module of the Comprehensive Antibiotic Resistance Database [23]. The program antiSMASH 7.0 [24] was used to investigate the presence of biosynthetic gene clusters. The presence of plant growth-promoting traits in the CWF10 strain was predicted using the PLant-associated BActeria web resource (PLaBAse)-based PGPr_finder program [25,26].

## Results and discussion

**Fig. 1.**
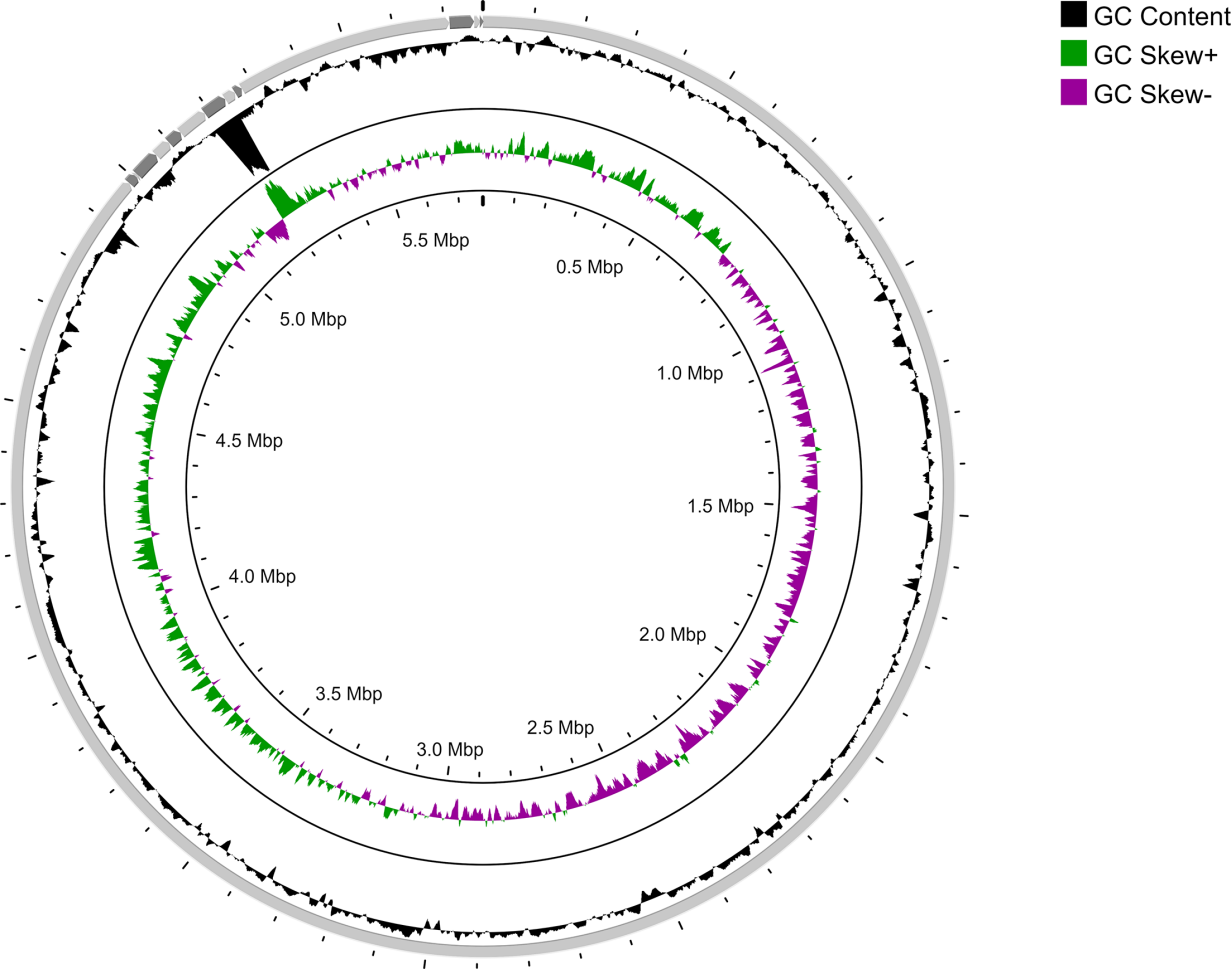
Circular genome map of *Azotobacter* sp. strain CWF10.

**Fig. 2.**
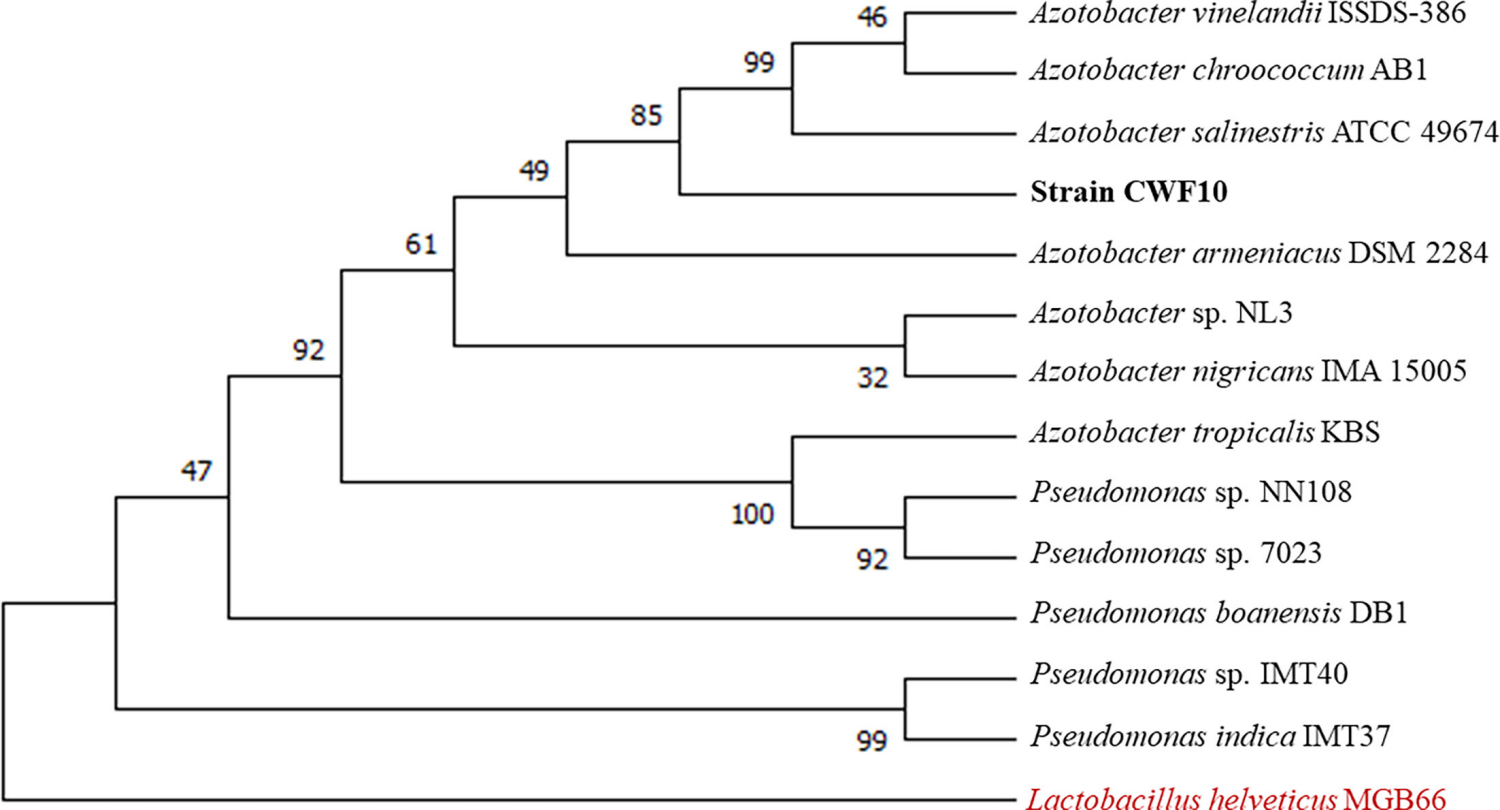
Phylogenetic analysis based on 16S rRNA sequence from maximum likelihood-based phylogenetic tree with the Tamura–Nei model (1000 bootstrap replicates, bold font marks investigated strain and red font as out group).

**Fig. 3.**
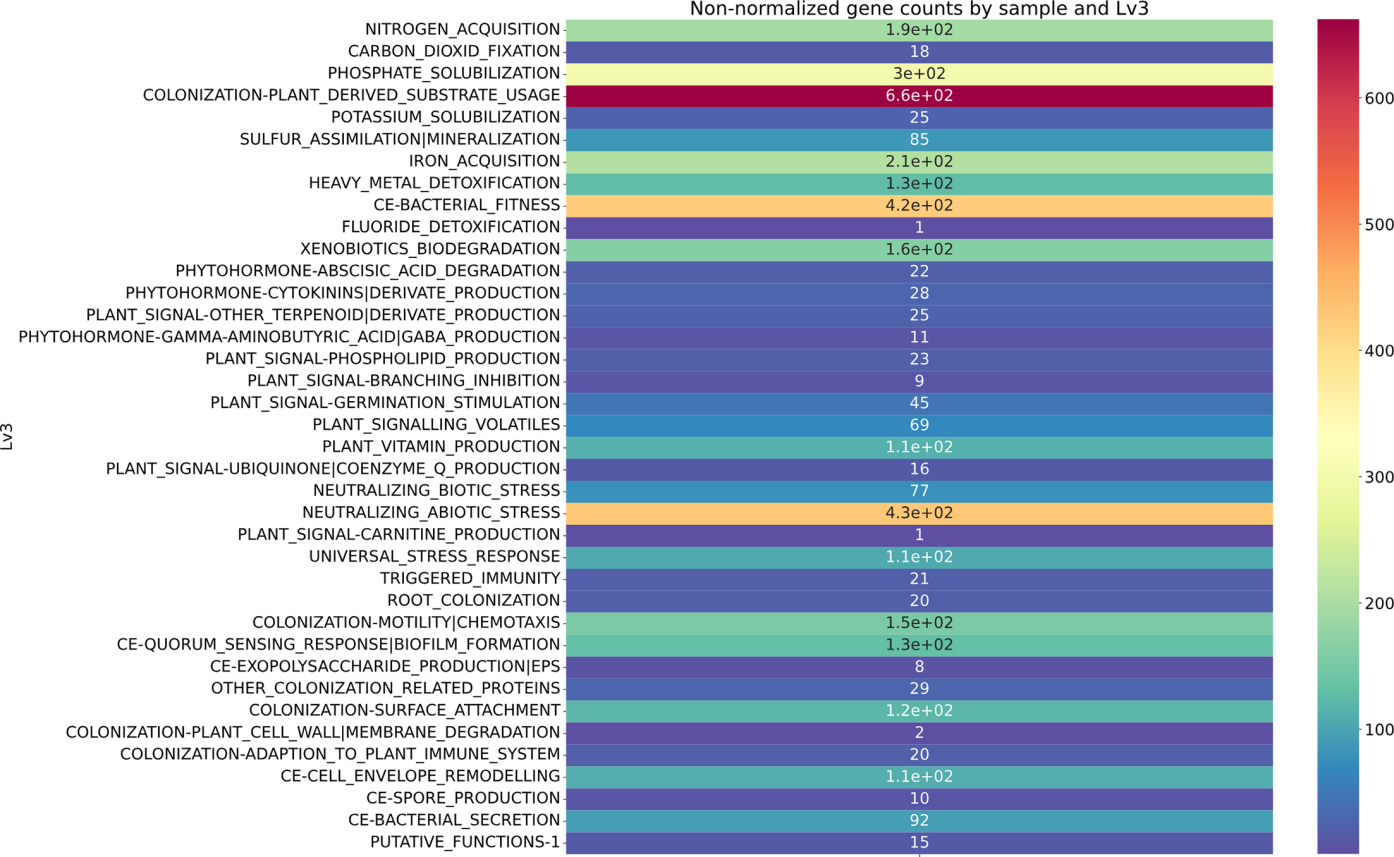
Plant growth-promoting gene categories and count from the identified genome of *Azotobacter* sp. strain CWF10.

The isolated bacterium CWF10 appears pale brown, slimy, produces a halo zone and forms cysts on nitrogen-free medium within 4–5 days, whereas, on nitrogen-rich Luria–Bertani medium, the bacterium grows overnight, and cells become aggregated and do not form any cyst (Fig. S2, available in the online version of this article). Genome sequencing of strain CWF10 resulted in the generation of 34 602 raw reads with 144 316 267 bp. *De novo* assembly with QC-ed reads achieved a coverage of 25X; 14 contigs were present and covered a length of 5 777 240 bp. The G+C content was calculated to be 65.09%, with the largest contig measuring 4 986 236 bp (Fig. 1). The completeness score of the genome was found to be 92.64%, reflecting an acceptable benchmark for a high-quality genome [27]. The statistics reflecting genome assembly are provided in Table 1. The draft assembly consists of 95 transfer RNAs, 19 ribosomal RNAs and 6710 coding sequences (Table S1). The strain possesses flagellar biosynthesis protein (*FlhB*, *FliP*, *FliQ* and *FliR*), basal-body rod protein (*FlgB, FlgC, FlgG* and *FlgF*), motor switch protein (*FliG* and *FliM*) and L, M, P ring protein and further support the genome level evidence of motility. The 16S rRNA (1535 bp)-based phylogenetic analysis revealed that strain CWF10 shares a phyletic lineage with *Azotobacter vinelandii* ISSDS-386 (99.33%), *Azotobacter salinestris* ATCC 49674 (99.39 %) and *Azotobacter chroococcum* AB1 (99.22%). This supports the taxonomic affiliation of CWF10 to *Azotobacter* (Fig. 2). The calculation of inter-genomic distance from pairwise comparison via FastME 2.1.6.1 also supports the taxonomic affiliation of strain CWF10 with *Azotobacter* (Fig. S1). The dDDH and ANI are particularly important for genome-wide comparison of two closely related taxonomic relatives based on shared orthologous gene clusters. ANI <95–96 % corresponds to dDDH value <70 %. Based on dDDH and ANIb calculations, it can be concluded that this strain represents a novel species of *Azotobacter* (Table 2); values were found to be below the acceptable threshold (ANI <95–96 %, dDDH <70 %) considered for species delineation [19,28,30]. The genome possesses information for heavy metal resistance, including copper (*CopA, CopB* and *MmcO*), arsenic (*Acr3*), cobalt–zinc–cadmium resistance protein (*CzcA* and *CzcB*) and nickel and cobalt resistance protein (*CnrA*). Interestingly, the genome encodes for arsenical pump-driving ATPase which is crucial for arsenic detoxification. Strain CWF10 is resistant to fluoroquinolone, tetracycline and diaminopyrimidine antimicrobial drug classes. The genetic blueprint of *Azotobacter* sp. strain CWF10 contains *abaQ*, *rsmA*, *adeF* and *qacG* genes linked to antimicrobial resistance. AntiSMASH results confirmed the presence of seven biosynthetic gene clusters in contigs 1 and 3. The presence of NRPS-independent vibrioferrin siderophore (100% similarity) in *Azotobacter* sp. strain CWF10 may help in iron acquisition during episodic fluctuations of metal availability. The isolated bacterium can putatively synthesize amphibactin and crochelin A siderophores, as reported previously [31,32]. The low-affinity vibrioferrin siderophore and the other two high-affinity siderophores together can combat iron starvation through the bucket brigade mechanism for continuous iron supply to the bacterium [33]. The genome also harbours carotenoid and type III polyketide synthase BGCs. The investigated strain encodes a wide array of plant growth-promoting genes, including nitrogen fixation and phosphate solubilization (Fig. 3). The genome possesses the complete pathway for nitrogen fixation, including nitrogenase molybdenum-iron protein (*nifDK*) and dinitrogenase reductase (*nifH*), as well as single copies of *PhoB, PhoR, PstA, PstB* and *PstS* for phosphorous metabolism. Phosphate-specific transporter and phosphate-import permease protein play a major role in the solubilization of inorganic phosphate and can make it bioavailable to plants. The investigated strain can putatively transform nitrate to ammonia due to the presence of *NasAB* and *NasBDE*. It also encodes for anthranilate synthase and anthranilate phosphoribosyltransferase, crucial for tryptophan biosynthesis and linked to auxin production [34]. The presence of chemotaxis proteins (*CheA* and *CheY*) and methyl-accepting chemotaxis proteins can regulate the phosphorylation process of the basal body and play a role in flagellar movement [35]. The genome also contains *XerC* and *XerD,* which play a putative role in rhizosphere colonization, along with 4-hydroxybenzoate transporter (*PcaK*). Aminodeoxychorismate lyase, crucial for folate biosynthesis, is present in the genome, indicating its importance towards plant growth. The bacterium can also biosynthesize trehalose through the maltooligosyl trehalose synthase enzyme and thus can help towards combat drought stress [36]. The rapid use of pesticides and fertilisers acts as a source of heavy metals, disrupting the functioning of soil microbiome, fertility and ecological balance [37,38]. *Azotobacter* spp. maintains soil stability, increases food crop production and is pivotal in facilitating availability of nutrients required by plants [39,40]. Strain CWF10 possesses a repertoire of metal resistance gene clusters along with other beneficial genes proving cues to environmental adaptation, which could lead to less accumulation of metals in the food chain. Nonetheless, genome-wide function-rich plant growth traits of *Azotobacter* sp. CWF10 suggest that it can augment agricultural productivity and need to be further evaluated based on robust on-field application. In-depth *in silico* investigation of *Azotobacter* sp. strain CWF10 indicates a potential new species with a broad range of plant growth-promoting traits and genetic signatures for bioremediation. This new isolate could be a potential candidate as biofertilizer to achieve regenerative climate-smart agriculture and improve agricultural soil health.

**Table 1.** Draft genome statistics of *Azotobacter* sp. strain CWF10

Genomic features	No. or length (bp)
G+C content (%)	65.09
No. of all contigs	14
No. of large contigs (>1000 bp)	14
Contig N50	4 986 236
Contig N90	433 960
L50	1
L90	2
Total length	5 777 240

**Table 2.** Comparison of *Azotobacter* sp. strain CWF10 with closely related species

Species	ANIb (%)	Distance	DNA–DNA hybridization (%)	G+C (%)
*Azotobacter salinestris*	94.66	0.0483	62	65.6
*Azotobacter vinelandii*	88.34	0.1038	38.7	65.7
*Azotobacter chroococcum*	90.39	0.1110	42	65.7
*Azotobacter beijerinckii*	87.45	0.0929	36.8	65.6

## Supplementary material

10.1099/acmi.0.000930.v4 Uncited Supplementary Material 1.

## Abbreviations

ANI: average nucleotide identity
dDDH: digital DNA–DNA hybridization
