# Supplementary material for: Insights into the genome of Azotobacter sp. strain CWF10, isolated from an agricultural field in Central India
Source: Access Microbiol. 2025 Jan 28;7(1):000930.v4. doi: 10.1099/acmi.0.000930.v4 (PMC12282048; doi:10.1099/acmi.0.000930.v4)

Table S1: Functional annotation statistics of *Azotobacter* sp. strain CWF10

**Features**	**Count**
Total number of coding sequences	6710
Predicted genes	3974
Duplicated genes	3456
Protein encoding genes with functional assignment	2548
Protein encoding genes without functional assignment	1426
tRNA	95
rRNA	19
Under presented genes	1354

Fig S1: Phylogenomic tree of *Azotobacter* sp. strain CWF10 derived from whole genome sequence

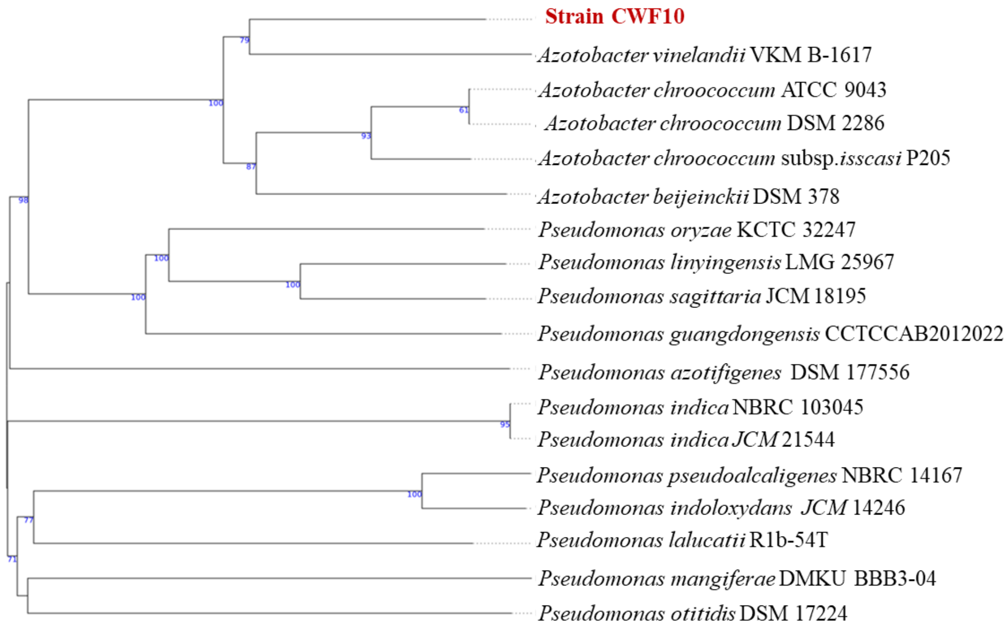

Fig S2: Colony and cell morphology of *Azotobacter* sp. strain CWF10 on nitrogen rich medium (A, C) and nitrogen free medium (B, D)

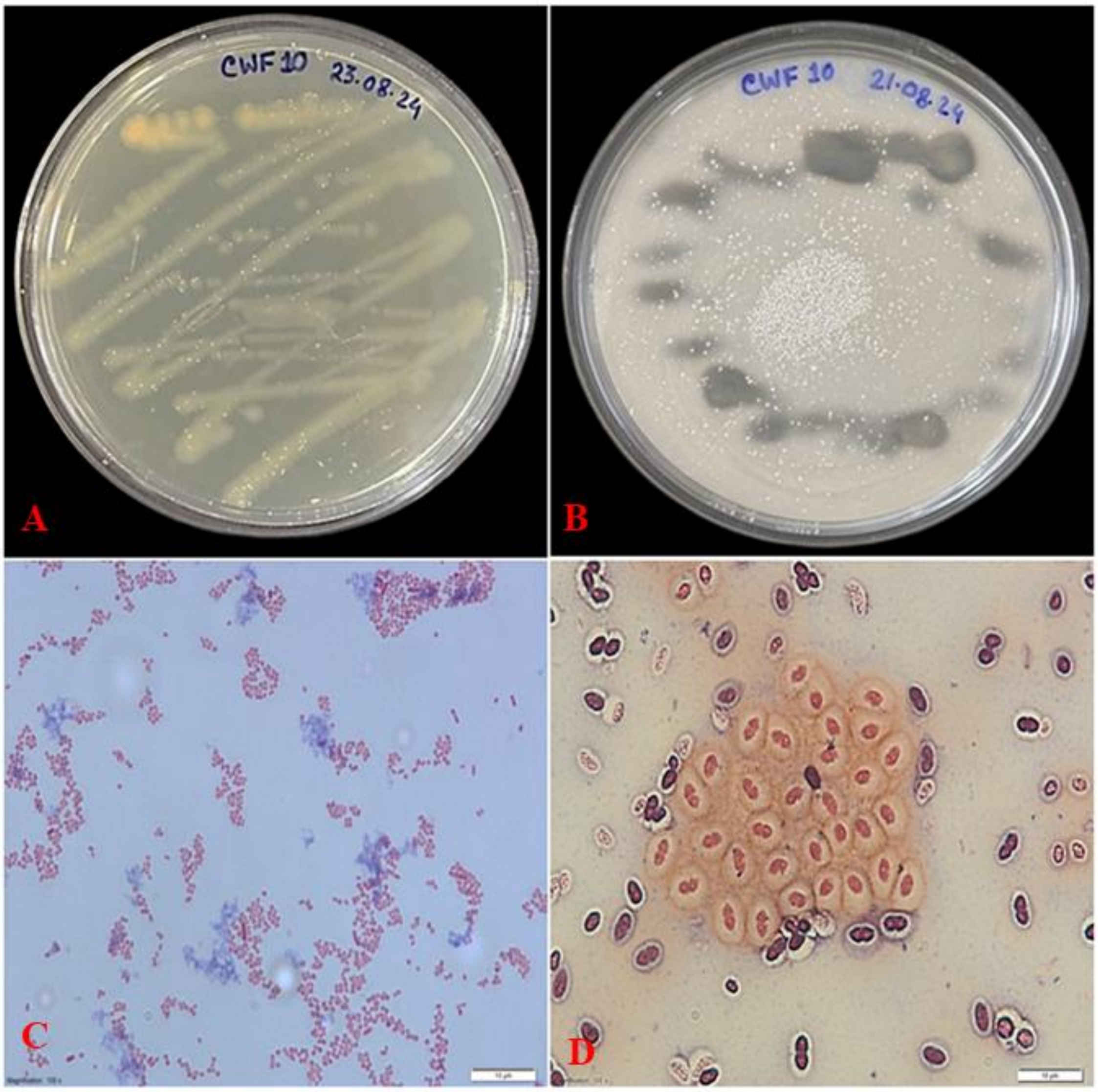

Supplement: Uncited Supplementary Material 1. [file acmi-7-00930-s001.pdf]
